# Effect of Allogenic Mesenchymal Stem Cell Injection on Functional Repair Outcomes Following Skeletal Muscle Laceration Injury

**DOI:** 10.3390/biomedicines13112810

**Published:** 2025-11-18

**Authors:** Raja Elina Ahmad, Abdul Halim Mokhtar, Mohamed Zubair Mohamed Al-Fayyadh, Hui Yin Nam, Atiqah Aziz, Azura Mansor, Tunku Kamarul

**Affiliations:** 1 Department of Physiology, Faculty of Medicine, Universiti Malaya, Lembah Pantai, Kuala Lumpur 50603, Malaysia; 2 Sports Medicine Unit, Faculty of Medicine, Universiti Malaya, Kuala Lumpur 50603, Malaysia; 3 National Orthopaedic Centre of Excellence for Research and Learning (NOCERAL), Department of Orthopaedic Surgery, Faculty of Medicine, Universiti Malaya, Kuala Lumpur 50603, Malaysia; zubair@um.edu.my (M.Z.M.A.-F.); namhy@utar.edu.my (H.Y.N.); 4 Tissue Engineering Group, National Orthopaedic Centre of Excellence for Research and Learning (NOCERAL), Department of Orthopaedic Surgery, Faculty of Medicine, Universiti Malaya, Kuala Lumpur 50603, Malaysia; eyqa@um.edu.my (A.A.); tkzrea@ummc.edu.my (T.K.); 5 KPJ Healthcare University, Lot PT 17010 Persiaran Seriemas, Kota Seriemas, Nilai 71800, Negeri Sembilan, Malaysia

**Keywords:** muscle injuries, muscle regeneration, mesenchymal stem cells, stem cell transplantation, wound healing

## Abstract

**Background**: Skeletal muscle laceration injuries remain a clinical challenge owing to limited and often delayed functional recovery. Surgical repair often fails to fully restore injured muscle, causing fibrosis and functional impairments. Mesenchymal stem cells (MSCs) represent a potential therapy due to their regenerative and immunomodulatory properties. However, their short-term regenerative effects in laceration injuries remain under-explored. **Objective**: We aim to evaluate the short-term effects of allogenic bone marrow-derived MSCs on skeletal muscle regeneration following laceration injury in rats. **Methods**: Sprague Dawley rats underwent laceration injury to the right gastrocnemius muscle and received local injection of either saline (n = 6) or allogeneic bone marrow-derived MSCs (2 × 10^6^ cells; n = 6) two weeks after injury. Muscle functional recovery was evaluated by measuring tetanic contraction force of the injured relative to the contralateral uninjured leg and compared among MSC-treated, saline-treated, untreated injured (n = 6), and intact control groups (n = 6) on days 7 and 14 post-treatment. Histological assessment of the treated muscle groups using Hematoxylin and Eosin and Masson’s Trichrome staining was conducted on day 7 post-treatment. **Results**: On day 7 post-treatment, MSC-treated muscle showed higher normalised force (96.8 ± 15.0%) than saline-treated (76.7 ± 4.6%) (*p* = 0.0393), but not untreated, muscle (83.1 ± 14.7%) (*p* = 0.2259). By day 14, the MSC-treated group exhibited significantly greater recovery of muscle force (110.8 ± 6.46%) than both the saline-treated (78.4 ± 6.47%) (*p* < 0.0001) and untreated groups (88.1 ± 3.41%) (*p* = 0.0001). Force recovery in the MSC-treated muscle was comparable to that in intact muscle (102.6 ± 10.4%) at both time points (*p* = 0.230). Supplementary histological analysis showed mild inflammatory cell infiltration, well-formed myoblasts, and a lower fibrosis index in MSC-treated muscle (29.30 ± 0.29%) compared with saline-treated muscle (31.77 ± 0.43%) (*p* < 0.0001) on day 7 post-treatment. **Conclusions**: Allogeneic bone marrow-derived MSC therapy is associated with enhanced repair of lacerated skeletal muscle over a short recovery period; however, larger studies with broader assessments are needed to confirm its potential clinical applicability.

## 1. Introduction

Traumatic injury to skeletal muscle is a common disabling problem that can be challenging to treat. Although a complete or near complete recovery of muscle function is expected to occur in less severe injuries such as contusions, full recovery to the pre-injury state may not be attained in severe cases such as laceration or those involving volumetric muscle loss [1]. In these cases where surgical repair is warranted, complete healing may be hampered by insufficient muscle regeneration or extensive fibrosis, which may lead to persistent functional deficits, increased morbidity, and, ultimately, reduced quality of life in affected individuals [2,3]. Laceration injuries normally occur as a result of direct assault on the muscle typically caused by sharp instruments or hard objects moving at a high velocity [4,5]. These injuries constitute a large number of traumatological cases seen in many developing countries, where criminal violence-related penetrating injuries or workplace injuries are often prevalent [6,7]. Indeed, in several previous reports, muscle injury that ultimately requires some form of surgical repair constitutes between 10 and 40% of all types of cases treated at the emergency department [8,9,10].

To date, the only established treatment for repairing lacerated muscles is suturing the opposing edges of the wounded epimysium, which results in closure of the external sheath covering the damaged muscle [11,12,13]. Suturing the transected muscle may facilitate muscle healing to a certain extent; however, the technique holds a risk of failure in achieving a complete functional recovery since it does not address the issue of complete opposition of the disrupted sarcomere in the myofibrils. The remaining myofibres may be incapable of bridging across gaps created by the injury. In addition, simple stitches could also lead to extensive scar tissue formation [14,15]. While suturing is the conventional surgical approach for muscle laceration repair, particularly in clinical settings, it primarily restores epimysial continuity and does not guarantee full alignment or regeneration of the underlying myofibres. In preclinical studies, especially in small animal models, suturing is technically challenging for standardised injuries of this size and depth, and may itself induce additional trauma or scarring at the repair site. Although skeletal muscle has a remarkable capacity to regenerate spontaneously after an injury due to activation of progenitor cells referred to as the satellite cells [16,17], the regeneration process is often interrupted and incomplete due to prolonged inflammatory reaction and fibrosis occurring at the damaged site [18,19]. This may ultimately compromise muscle function.

In recent years, there has been a resurgence of interest in exploring the potential use of cell therapy such as myogenic progenitor cells (myoblasts or satellite cells) [14,15,16,17,18,19,20] and mesenchymal stem [21,22,23,24] or stromal cells [25,26,27,28,29,30] to repair damaged muscle. The use of local resident muscle cells as donors has several inherent limitations, which includes insufficient number of cells available for extraction, limited expansion potential ex vivo, and limited migration capability of the cells [31]. On the other hand, multipotent stem cells particularly from allogenic sources may present as a more attractive alternative cell source in view of their remarkable regenerative potential and immunomodulatory properties. Mesenchymal stromal cells (MSCs) currently constitute the most frequently used cell type in advanced treatment of various disorders [32,33]. These cells can be conveniently derived from non-muscle sources such as bone marrow [26], adipose [34,35], and umbilical cords [36,37]. Another advantage of using these cells is the fact that they can be expanded with sufficient quantities ex vivo and may be able to support myofibre regeneration either by directly differentiating into myogenic cells or fusing with existing skeletal myotubes [38]. Alternatively, their paracrine effect on the satellite cells residing between the sarcolemma and the basal membrane within the vicinity of the injured site have also been implicated in hastening the repair process. The effect of bone marrow-derived MSC transplantation on skeletal muscle regeneration has been previously assessed in several experimental studies [25,29,39,40,41]. However, there is still lack of reports on the outcomes of MSC treatment for muscle repair, particularly those involving laceration injury, as previous studies have mainly focused on blunt or crush injury models. The role of MSCs in myofibre regeneration after laceration injuries thus warrants further exploration.

It remains to be determined whether the transfer of MSCs into lacerated muscle leads to improved repair outcomes when assessed within a short period after treatment. If MSC treatment is proven to be effective in expediting functional recovery of severely damaged muscle, such an intervention would be favourable to allow for early return to activity in affected individuals. Therefore, this study aimed to evaluate the effects of allogenic bone marrow-derived MSCs on skeletal muscle regeneration following laceration injury in rats 7 and 14 days post-treatment. We hypothesise that allogeneic bone marrow-derived MSC (BM-MSC) transplantation improves functional recovery of lacerated skeletal muscle within a short period of time after treatment.

## 2. Materials and Methods

### 2.1. Animals

Female Sprague Dawley rats aged between 8 and 10 weeks, weighing between 200 and 250 g, were used in this study. The animals were obtained from the Animal Experimental Unit, Universiti Malaya. All animal experiments were conducted according to the guidelines for animal handling and welfare of the University of Malaya. The present study has been approved by the Universiti Malaya ethics review committee for animal research [OS/13/12/2010/RZHRY(R)].

A priori power analysis was performed using G*Power version 3.1.9.7. For a one-way ANOVA with three groups, assuming a large effect size (f = 0.40) [42], significance level α = 0.05, and power (1–β) = 0.80, the required sample size was calculated to be 24 animals (6 per group). Therefore, the group size used in this study was deemed sufficient to detect large treatment effects.

The animals were subjected to muscle laceration injury and divided into 3 distinct groups: (1) no treatment (n = 6); (2) saline treatment (n = 6); and (3) MSC treatment (n = 6). An additional group consisting of 6 rats was used as a positive control, where no laceration was performed, nor any treatment given. Data from this group was used as the baseline data representing normal rats with intact muscles. Therefore, a total of 24 rats (N = 24) were used in this experiment.

### 2.2. Animal Model of Muscle Laceration

**Figure 1:**
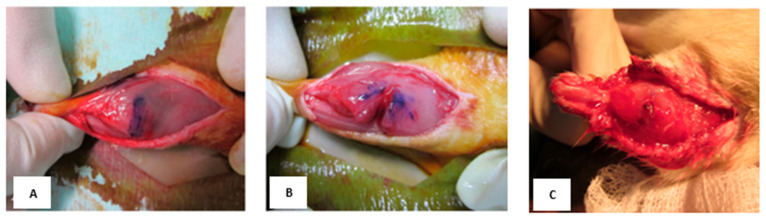
Macroscopic appearance of an intact and lacerated muscle. (**A**) Intact muscle marked for wound creation; (**B**) laceration injury model; (**C**) healed muscle at 7 days post-treatment with BM-MSCs.

Rats were anaesthetised using an intraperitoneal injection of 50 mg/kg (100 mg/mL) of ketamine hydrochloride and 5 mg/kg (20 mg/mL) of xylazine hydrochloride. After 15 min, the animal was placed in a prone position, and a longitudinal skin incision was made to expose the right gastrocnemius muscle (Figure 1). A laceration injury model was created by dissecting the muscle in a standardised manner, i.e., at the point of 60% of the muscle length from the distal insertion, through 75% of the width and 50% of the thickness. The fascia was closed using Vicryl absorbable sutures, and the skin overlaying the muscle was closed with 4-0 black silk sutures. The laceration was only performed on the right leg, whilst the left leg served as the individual control for the respective animal.

### 2.3. Rat Bone Marrow-Derived Mesenchymal Stromal Cell (BM-MSC) Preparation and Transplantation

BM-MSCs were obtained from the femur and tibia of 24 rats which were sacrificed 4 weeks prior to the transplantation procedure. BM-MSCs were pooled for use in all rats in the MSC-treated group. BM-MSC isolation was carried out according to the methods previously described in our previous publications [43,44]. Briefly, the mesenchymal stromal cells were isolated from the tibia and femur of the euthanized rats. The bones were stored in 1× phosphate-buffered solution (PBS) with 1% penicillin/streptomycin (Invitrogen-Gibco, Carlsbad, CA, USA). Isolation of the rat BM-MSCs was performed using density centrifugation with Ficoll-Paque PREMIUM (density 1.077 g/mL) (Amersham Biosciences, Uppsala, Sweden) to isolate the mononuclear cell layer. The cells were then washed with PBS and suspended in culture medium Dulbecco’s Modified Eagle Low Glucose with 1% antibiotic-antimycotic solution and 10% foetal bovine serum (Invitrogen-Gibco, USA) and seeded on culture flasks. The flasks were incubated at 37 °C in a humidified atmosphere containing 5% CO_2_. The culture medium was replaced every 3 days, and adherent cells were left to grow on the flask surface. The adherent cells were serially passaged and expanded up to passage 2 before being used for transplantation. Transplantation of the BM-MSCs into damaged muscle was performed two weeks after creating the laceration injury. The BM-MSCs (2 × 10^6^ cells) were trypsinised and re-suspended in 500 µL of 0.9% saline solution before being injected into the injured gastrocnemius muscle of rats in the MSC-treated groups. For the saline group (sham treatment), a similar volume of saline without the addition of any cells was injected into the injured muscle in a similar manner. The 2-week interval was selected to allow the initial inflammatory phase of muscle healing to subside, as early transplantation during peak inflammation has been associated with poor survival (<10%) of transplanted myogenic precursor cells [45].

### 2.4. Physiological Test: Muscle Force Measurement After Tetanic Contraction

**Figure 2:**
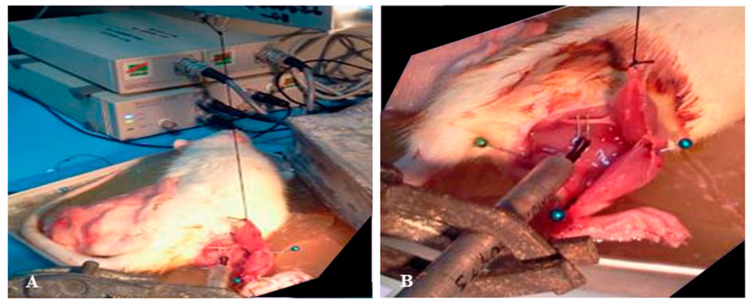
Physiological test models. (**A**) A thread was tied around the edge of the incised Achilles tendon and hooked up to the force displacement transducer; (**B**) the rat’s hind limb was pinned down to the base board to immobilise the knee and ankle joint.

The muscle force recording measurement was obtained using the Power lab data acquisition system (ADInstruments, Bella Vista, Australia) on days 7 and 14 post-treatment. Before the test, the rats were anaesthetised and the wound re-opened. The Achilles tendon was cut to free the distal end of the gastrocnemius muscle. A piece of thread was tied around the edge of the incised Achilles tendon, and the opposite end of the thread was hooked up to the force displacement transducer, which was connected to a recording device (Figure 2A). The rat’s hind limb was firmly pinned down to the base board to immobilise the knee and ankle joint (Figure 2B). The sciatic nerve was identified and carefully exposed. A two-pronged electrode was placed in direct contact with the sciatic nerve, providing electrical stimulation. Care was taken throughout the procedure to ensure that the nerve–muscle connection remained intact. Throughout the course of the experiment, the animal was kept warm at 37 °C by using a heating lamp, and the exposed muscle was kept moist by continuously bathing it with warm ringer solution. The muscle surface temperature was monitored constantly via a thermometer. The signal generated from the force recording device was initially calibrated against pre-determined weights according to the manufacture protocol, which allowed the measurement of muscle force or tension to be expressed in terms of weight displacement, i.e., gram-tension (g). Prior to measurement of the tetanic contraction, the experiment protocol was optimised for the appropriate muscle resting length (L_0_) and stimulation voltage in each animal. Briefly, the muscle length was initially adjusted at an increment of 0.3 mm until a maximum amplitude of a single twitch was observed. At this optimal length (L_0_), the stimulus strength was then increased by 0.5 V from an initial value of 1 V until a maximum amplitude of muscle twitch was achieved. The selected voltage of stimulation was doubled to ensure supra-maximal stimulation that would induce recruitment of all motor units. At this optimal voltage, the stimulation frequencies were then increased from 25 Hz up to 200 Hz until a plateau in force measurement was clearly observed. This was taken as the measurement of the maximal force of tetanic contraction for the individual rat. Our preliminary study indicated that maximal force upon tetanic contraction was elicited at a frequency of stimulation of 100 Hz. Hence this frequency was selected to induce tetanic contraction in all animals. Once testing of one leg was completed, the surgical procedure and muscle force recording were repeated for the contralateral leg. To minimise muscle fatigue and avoid confounding effects on subsequent measurements, a 15 min interval was allowed between testing of the injured and uninjured muscles, ensuring that contractile assessments accurately reflected the physiological status of each limb. The sequence of testing (injured vs. non-injured leg) was randomised to minimise selection bias related to the order of testing. All force measurements were normalised to that of the uninjured leg of each animal. All muscle force measurements were performed by an investigator who was not involved in the surgical or transplantation procedures, and the testing order of injured versus uninjured limbs was randomised. For histological analysis, slides were coded and assessed without knowledge of the treatment group to minimise observer bias.

### 2.5. Histology

To complement the primary assessment of muscle functional recovery, a supplementary histological evaluation was performed to provide supportive insight into the underlying tissue morphology. After the completion of the physiological testing to determine the force of tetanic muscle contraction, the rats were sedated, euthanized, and sacrificed. The area around the lacerated muscle was carefully examined (Figure 2) and the tissues were subsequently subjected to histological analysis. The gastrocnemius muscles were isolated and frozen in 2-methylbutane pre-cooled in liquid nitrogen. These samples were fixed in 10% buffered formalin for 48 h and decalcified in 10% formic acid. The specimens were processed for histological slides, embedded in paraffin wax, and sectioned at a thickness of 5 µm. The slides were stained using Hematoxylin and Eosin (H&E) staining and Masson’s Trichrome for histology. The histological images were analysed to evaluate the extent of inflammation, tissue disruption and necrosis, fibrosis, collagen fibres, cellular proliferation, and overall tissue re-organisation.

Masson’s Trichrome-stained sections were qualitatively examined to evaluate overall tissue morphology and collagen deposition, where muscle fibres were stained red and collagen fibres stained blue. Colour distribution analysis was performed using ImageJ software (version 1.54p, National Institutes of Health, Bethesda, MD, USA) to quantify the relative intensity of red and blue colours in histological images. Colour images (JPEG or TIFF format) were opened in ImageJ, and the RGB colour components were measured directly from the image. The “Colour Histogram” function was used to obtain the mean intensity, standard deviation (SD), and total pixel area for each colour channel (red, green, and blue). For each image, a uniform grid was applied to generate 20 equal-sized boxes, of which 16 boxes were randomly selected using an online random number generator for measurement to ensure representative sampling. The red-to-blue (red/blue) ratio was calculated for each box as the mean red intensity divided by the mean blue intensity, and the SD of the ratios was determined accordingly. In addition, the fibrosis index (%) was calculated according to the method described by Tanano et al. (2023) [46] using the following formula:

Fibrosis Index (%) = [Mean Blue/(Mean Red + Mean Blue + Mean Green)] × 100

Six biological samples (n = 6) were analysed, and data are presented as mean ± SD across all selected regions. Statistical comparisons between experimental groups were conducted using a one-tailed unpaired (independent) *t*-test.

### 2.6. Statistical Analysis

**Figure 3:**
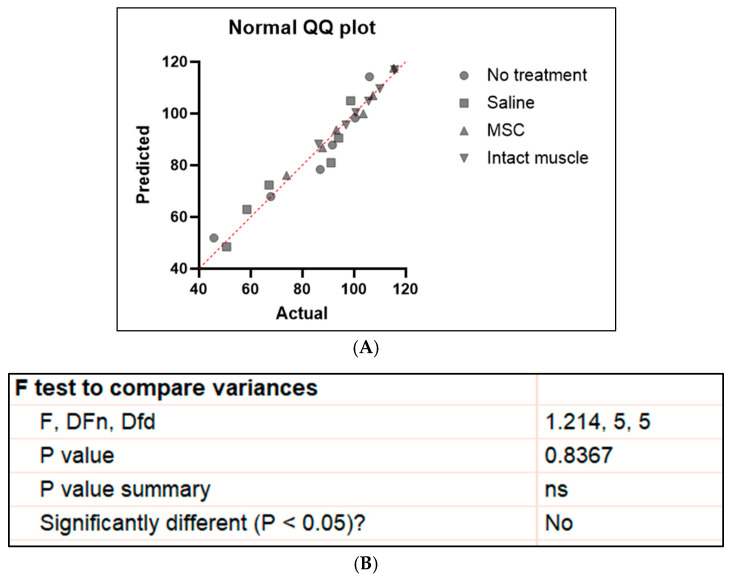
(**A**) Q–Q plots showing no major deviations from normality. (**B**) Output from Levene’s test (F-test). Homogeneity of variances was verified based on the results showing equal variances among groups (*p* = 0.8367). ns denotes non-significant results.

Statistical analyses were conducted using GraphPad Prism version 10 (GraphPad Software, Boston, MA, USA). Data normality was confirmed using the Shapiro–Wilk test (all *p* > 0.05), and Q–Q plots showed no major deviations from normality (Figure 3A). Homogeneity of variances was confirmed using Levene’s test (*p* > 0.05) (Figure 3B). Therefore, parametric analyses were applied. For comparisons among three groups, one-way ANOVA was used. Results are presented as mean ± SD, and *p* < 0.05 was considered statistically significant. To provide more reliable estimates and inferences in view of the small sample size in this study, a bootstrap analysis was further performed. To perform the bootstrap analysis, the original data were placed in cells (n = 6). Random indices were generated in another column using the formula = RANDBETWEEN (min sample row, max sample row) to randomly select data points with replacement. These indices were then used in the formula = INDEX ($selected_cells_range$, RANDBETWEEN (1,6)) to create resampled datasets of equal size. For each bootstrap sample, the mean was calculated, and this process was repeated 10,000 times by dragging the formulas or using a macro. From the 10,000 bootstrap means obtained, the average value (bootstrap mean) and the 2.5th and 97.5th percentiles were computed to determine the 95% bootstrap confidence interval.

For within-subject comparisons of muscle force between the right and left sides of intact muscles as well as differences in the mean blue/red ratio and fibrosis index, paired *t*-tests were applied. A *p*-value of <0.05 was considered statistically significant

## 3. Results

**Figure 4:**
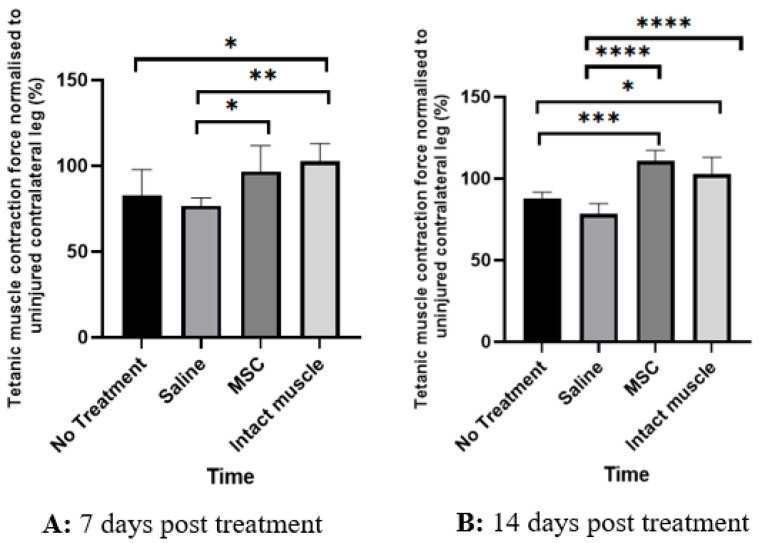
Tetanic muscle contraction normalised to the contralateral uninjured leg (%). Comparison of muscle force recovery of injured muscle relative to contralateral normal muscle among the untreated, saline-treated, MSC-treated, and intact muscle groups on day 7 (**A**) and day 14 (**B**) post-treatment, following a 2-week delay after injury. Data are presented as mean ± SD (6 subjects per group). Statistical significance among groups was determined using one-way ANOVA followed by Tukey’s post hoc test. * *p* < 0.05, ** *p* < 0.01, *** *p* < 0.001, **** *p* < 0.0001 compared to the indicated groups.

Table 1 shows the measurement of force of tetanic isometric contraction, expressed as weight displacement (in grams) between the right and left intact gastrocnemius muscle within the same animal. As seen in Table 1, despite both muscles being intact and originating from the same animal, a slight variation in force production was observed between the two legs, with the right leg tending to be dominant on average; however, this difference was not statistically significant. When expressed as the ratio of right-to-left leg force production, this variation was normalised, as reflected by a mean ratio of 102.6 ± 10.4%. The tetanic contraction force in grams as well as the ratio of muscle force between the injured (right leg) and uninjured (left leg) muscle across the study groups at day 7 and 14 post-treatment is shown in Table 2 and Table 3, respectively. Comparison of the mean ratio of tetanic contraction force between the injured (R) and uninjured (L) leg across the study groups after treatment is depicted in Figure 4. The ratio (in percentage) emphasises the degree of functional recovery relative to the animal’s own intact leg. As seen in Figure 4A, on day 7 post-treatment, the mean ratio of tetanic contraction force normalised to the contralateral uninjured muscle was significantly higher in the MSC-treated group than in the saline-treated group (*p* = 0.0393, Cohen’s d = 1.68, 95% CI = −39.40 to −0.80). No significant difference was observed between the saline-treated and untreated groups (*p* = 0.7902, Cohen’s d = 0.54, 95% CI = −12.90 to 25.70) or between the MSC-treated and untreated groups (*p* = 0.2259, Cohen’s d = 1.15, 95% CI = −33.00 to 5.60). As expected, the normalised muscle force ratio was significantly lower in the saline-treated (*p* = 0.0063, Cohen’s d = 2.17, 95% CI = −45.20 to −6.60) and untreated groups (*p* = 0.0471, Cohen’s d = 1.63, 95% CI = −38.80 to −0.20) compared with the intact-muscle group.

On day 14 post-treatment (~30 days post-injury), the MSC-treated group demonstrated a significantly higher mean normalised tetanic contraction force ratio compared with both the saline-treated (*p* < 0.0001, Cohen’s d = 4.55, 95% CI = −43.92 to −20.88) and untreated groups (*p* = 0.0001, Cohen’s d = 3.18, 95% CI = −34.22 to −11.18). The results in the MSC group were comparable to the intact-muscle group (*p* = 0.2241, Cohen’s d = 1.15, 95% CI = −3.32 to 19.72). The saline-treated group showed no significant difference from the untreated group (*p* = 0.1187, Cohen’s d = 1.36, 95% CI = −1.82 to 21.22), but its normalised force ratio remained significantly lower than that of the intact-muscle group (*p* < 0.0001, Cohen’s d = 3.40, 95% CI = –35.72 to −12.68).

**Figure 5:**
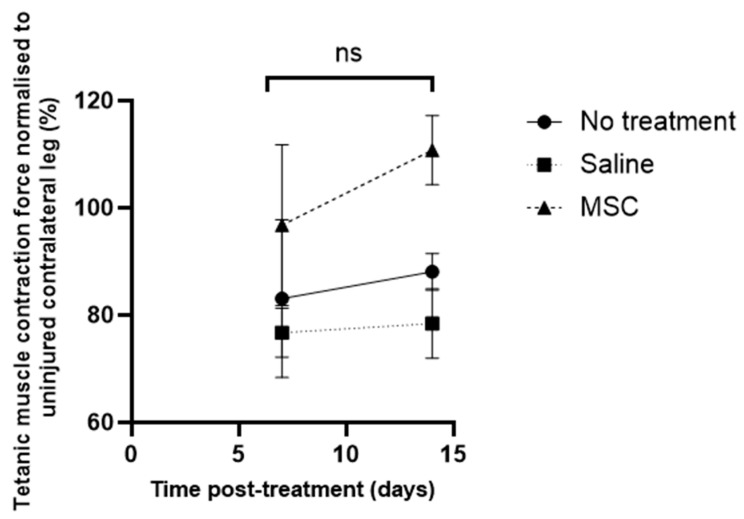
Tetanic muscle contraction force of injured muscle normalised to the uninjured contralateral leg (%) measured on day 7 and day 14 post-treatment. Data are presented as mean ± SD (6 subjects per group). Statistical comparisons among groups at each time point were performed using one-way ANOVA followed by Tukey’s post hoc test. A *p*-value < 0.05 was considered statistically significant; ns denotes non-significant results.

Intra-group comparisons between day 7 and 14 revealed that normalised muscle force tended to gradually increase further by day 14 post-treatment in all study groups; however, the temporal increments were not statistically significant (Figure 5).

**Figure 6:**
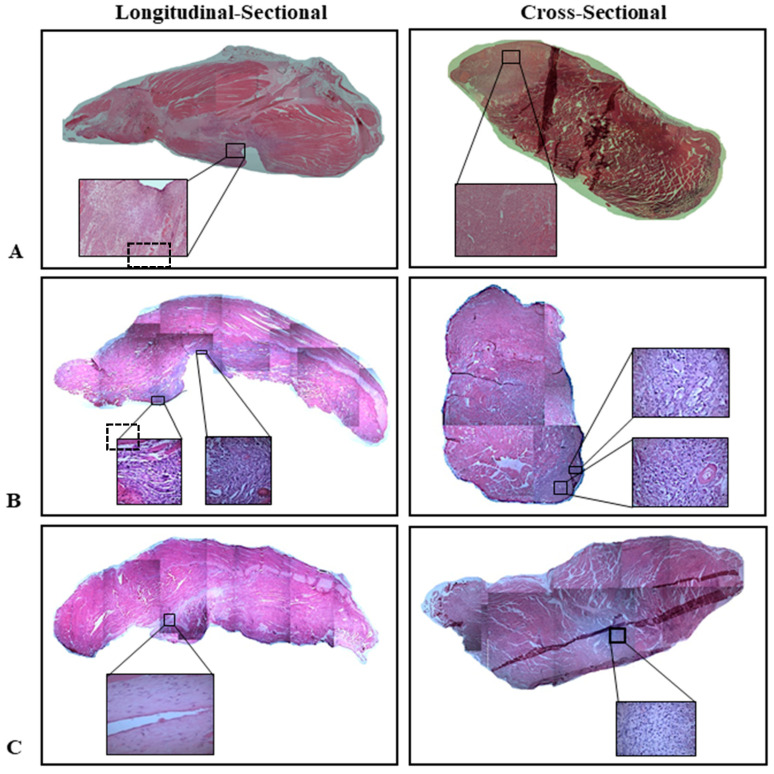
Representative longitudinal and cross-sectional H&E staining images of rat gastrocnemius muscle at 7 days. (**A**) Injured untreated; (**B**) saline-treated; (**C**) MSC-treated. Magnification 200×.

**Figure 7:**
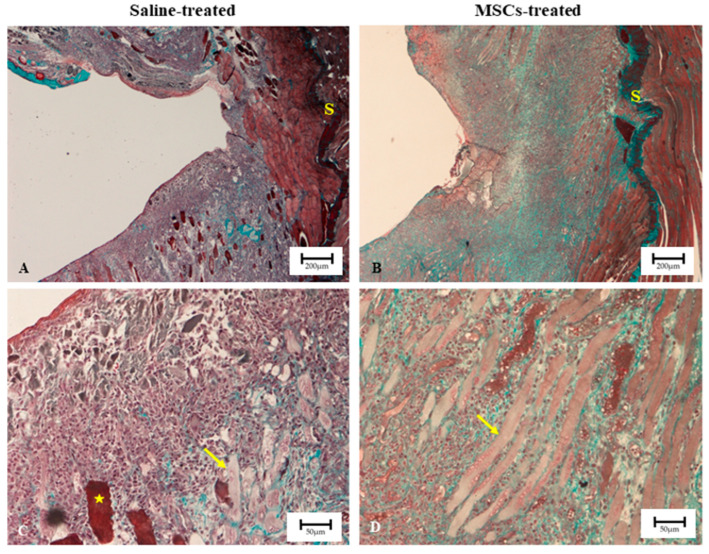
Photomicrographs of Masson’s Trichrome stains of muscles at injury site in rat 7 days after treatment with MSCs and saline. (**A**,**C**) Saline-treated; (**B**,**D**) MSC-treated muscles. MSC-treated images showing much reduced depth of the wound, increased thickness of muscle tissue to the left of the intramuscular septum (S) and appearance of well-formed myoblasts (arrow), while saline-treated images showed more inflammatory cells infiltration, disorganised extracellular matrix and presence of degenerating myofibres (asterisk symbol). Scale bar: (**A**,**B**) 200 µm; (**C**,**D**) 50 µm.

Figure 6 shows the representative longitudinal images of H&E staining of rat gastrocnemius muscles from the four study groups 7 days post-treatment. Masson’s Trichrome staining of the injured muscle showed that, in general, the incision areas in both groups were free from debris (Figure 7). In the saline-treated group (Figure 7C), some degenerating muscle fibres (indicated by yellow asterisks) were still observed, along with dense cell infiltration on both sides of the incision site. In contrast, in the MSC-treated group, both sides of the incision were largely occupied by actively forming myoblasts (shown in yellow arrow) (Figure 7D). Given that the incisions were performed at similar regions and comparable depths, it was notable that the MSC-treated muscles (Figure 7B) exhibited better recovery, as evidenced by a reduced wound depth and increased thickness of muscle tissue to the left of the intramuscular septum (labelled with S) in the MSC-treated muscles. In addition, at higher magnification (Figure 7D), inflammatory and interstitial cell infiltration was mild, and the presence of well-formed myoblasts was apparent in MSC-treated muscle. Further analysis of the Masson’s Trichrome-stained sections (at higher magnification) using ImageJ showed that, among the injured muscle groups, MSC-treated samples exhibited a relatively lower proportion of fibrotic area compared with the saline-treated group (Table 4).

## 4. Discussion

This study presents preliminary evidence on the effect of short-term allogeneic bone marrow-derived mesenchymal stem cell (MSC) application on regeneration capacity of the gastrocnemius muscle following laceration injury. The primary goal was to explore a potential therapeutic strategy that could improve functional recovery after traumatic muscle injuries and thus facilitate a more rapid return to work of the affected individuals. In line with this objective, muscle performance was evaluated as the principal outcome, based on contractile force measurements following tetanic electrical stimulation (expressed as the percentage of force restored relative to the contralateral uninjured muscle). A complementary histological examination was also conducted to provide additional supportive insight into tissue-level changes associated with the observed functional recovery. A two-week period was allowed to elapse following injury before treatment with either MSCs or saline, with the aim of allowing the initial inflammatory phase to subside and thereby creating a more favourable environment to support MSC viability and engraftment. Several key findings emerged that may offer preliminary insight into the therapeutic potential of MSCs in the short-term repair of muscle laceration. On day 7 post-treatment, the recovery of muscle force was significantly greater in the MSC group compared with saline treatment, although it did not differ from the untreated group, suggesting that the beneficial effects of MSCs are detectable early but may initially be modest. By day 14; however, a clear and statistically significant improvement in normalised tetanic muscle contraction was observed in the MSC group compared with both the untreated and saline-treated groups, indicating a more robust therapeutic effect. Our study also demonstrated no significant change in muscle contractile performance between day 7 and day 14 post-intervention within each group, suggesting that functional recovery generally progressed very slowly in all animals over this interval, irrespective of treatment. To complement the primary functional recovery findings, histological analysis of muscles subjected to a comparable extent of injury demonstrated enhanced regeneration in the MSC-treated group compared with the saline-treated (sham) group, as evidenced by less pronounced inflammatory changes, the presence of well-formed myoblasts, and a relatively lower fibrosis index.

The effect of mesenchymal stem cell transplantation on functional regeneration of injured muscle has been investigated in previous studies mostly in the context of contusion injury. This type of closed injury typically causes localised fibre disruption while preserving overall tissue continuity. It is relevant for modelling contusion trauma, such as bruises or sprains, which are likely more commonly encountered clinically; however, the extent of injury is generally less severe, as the lesion often does not extend into the deeper tissue layers. In this study, we focused on laceration injury because it represents one of the most prevalent forms of trauma in this region and is also clinically relevant, as skeletal muscle laceration may also occur as part of surgical procedures, for example, following tumour resection. This type of open injury involves a more extensive structural disruption, thereby pose a great challenge for functional regeneration. Moreover, reports on the effects of MSCs on the repair of lacerated muscles remain limited. Laceration injury involving muscle belly is a challenging lesion to repair because of the technical difficulty of implementing reliable and optimal suture methods, which may lead to a high probability of clinical failure [47].

The results of muscle functional outcomes in the present study showed that MSC treatment led to an approximately 26% improvement in the muscle force recovery relative to the contralateral uninjured leg compared with the saline-treated group on day 7 post-treatment (3 weeks after injury), which is consistent with the histological findings. The functional improvement was further accentuated to 41% on day 14 post-treatment (4 weeks after injury). We did not observe a significant difference in the muscle force recovery between the MSC-treated and the untreated muscle on day 7 post-treatment, which was unexpected. This could partly reflect the relatively small sample size in this study, where greater variability in measurements could have masked the true differences between the two groups. An alternative explanation for this observation is that spontaneous healing may have occurred to a greater extent within the initial 2-week period after injury in the untreated group, thus reducing the measurable impact of MSC treatment on muscle functional recovery. Delaying treatment for 2 weeks after injury was intended to improve MSC viability by allowing the initial inflammatory phase to subside, as the cell survival rate has been shown to be less than 10% when transplantation of myogenic precursor cells is conducted too early, such as on the fifth day of trauma [45]. Nevertheless, this strategy may warrant reconsideration in the design of future studies, as our findings raise the possibility that substantial spontaneous healing may have already occurred during this period, resulting in near-complete restoration of muscle strength and potentially masking any additional functional benefit attributable to MSC therapy. A similar 2-week interval between defect creation and MSC injection was reported in a previous study by Chiu et al. [28]; however, a more severe muscle contusion injury model was used in that study, which likely explains the more pronounced improvement in tetanic muscle strength in the MSC-treated compared to the control group in that study. Our results showed that contraction force of the injured gastrocnemius muscle in MSC-treated animals had almost fully recovered to the level of the uninjured contralateral leg (96.8 ± 15.0%) as early as day 7 post-treatment (3 weeks after injury) and exceeded that of the uninjured contralateral leg (110.8 ± 28.1%) by day 14 post-treatment (4 weeks post-injury). Although our histological findings demonstrated the presence of regenerating myofibres on day 7 post-MSC treatment, it is a bit unusual to expect complete restoration of muscle contractile strength at such an early stage. The extent of force recovery in our study was notably higher than that reported in previous studies [48,49,50,51]. The possibility that natural healing during the 2-week delay before intervention supplemented the effects of MSCs could partly account for this. It is also plausible that the laceration injury created in this study may not have been very substantial. If the injury was relatively modest, with clean sharp wound edges, natural healing of the muscle during the 2-week period before MSC injection could have also contributed to the recovery of muscle function observed in this group. A previous study using a laceration injury model involved a more substantial injury with volumetric muscle loss [42]. Our study indicated that the therapeutic impact of MSCs on muscle force recovery was most evident on day 14 post-treatment. Overall, the results of physiological measurements of muscle force recovery upon tetanic contraction in the present study generally corroborate the collective evidence from previous studies adopting both laceration and crush injury models, suggesting that MSCs have the capacity to enhance and restore skeletal muscle functional repair [25,26,29,42,48,51,52]. It is important to recognise that previous studies have reported variable efficacy of MSCs depending on whether they were delivered directly into the injury site, systemically, or in combination with biomaterial scaffolds, with each approach potentially influencing cell survival, engraftment, and paracrine activity [25,42,53]. Likewise, the cell dose appears to be a critical determinant, as higher doses have been associated with improved functional recovery in some studies [35]. Future studies exploring how different delivery methods, timing, and dosages of MSCs could impact both histological and functional recovery following skeletal muscle injury are highly valuable.

Although not the primary focus of the study, the supplementary qualitative histological assessment revealed that injection of BM-MSCs into lacerated muscle improved muscle healing as reflected by reduced wound depth and the presence of well-formed myoblasts in the MSC-treated group. Furthermore, quantitative analysis of the Masson’s Trichrome-stained sections revealed a relatively smaller area of fibrosis in the MSC-treated group compared with the saline-treated group. Although no comparison of fibrosis index was made between the two treatment groups and the intact muscle group, it can be reasonably expected that intact (uninjured) muscle demonstrates almost negligible collagen deposition or fibrosis under physiological conditions. Binder Markey et al. reported that intact muscle, comprising myofibres with their associated endomysium and perimysium but lacking large connective tissue structures, contains collagen levels approximately two orders of magnitude (i.e., about 100-fold) lower than those found in dense connective tissues such as internal tendons and aponeuroses [54]. Although that study did not directly assess fibrosis, the finding underscores that normal uninjured muscle has very minimal and almost negligible collagen content. Therefore, the increased collagen accumulation observed in the present study can reasonably be interpreted as fibrosis secondary to injury.

Our histological findings align with previous studies albeit at different time points of assessment. Natsu et al. demonstrated that transplanted BM-MSCs promoted maturation of myofibres histologically 1 month following laceration injury [48]. In another study involving large defect creation, Merritt et al. also showed higher cellularity and denser population of desmin (muscle-specific cytoskeletal proteins) and myogenin-positive nuclei around the defect site injected with MSC+ extracellular matrix (ECM) compared with ECM alone after 42 days of recovery from the injury, indicating increased numbers of regenerating myofibres growing within the implant induced by the MSC treatment [42]. However, it was unclear from the data whether the MSCs directly engrafted or merely created an environment that enhanced regeneration. Winkler et al. evaluated the effect of immediate MSC transplantation compared with transplantation 7 days after trauma in a blunt crush muscle injury model [49]. The results were compared for uninjured and saline-treated injured muscles. Histological analyses 4 weeks after injury showed an increase in regenerating small myofibres in all injured muscles (MSCs and saline-treated group) compared to the pooled uninjured controls; however, there were no significant differences in the myofibre diameters and extent of collagenous fibrotic tissue deposition between the MSC-treated and saline-treated injured muscles [49]. The transplanted GFP-labelled MSCs were detected up to 4 weeks after injection, mainly residing within the interstitial compartment of the muscle; however, differentiation of the MSCs to myoblasts or other type of cells, or their fusion with myofibres, could not be confirmed morphologically. A more recent study that evaluated the effect of bone marrow-derived MSCs on muscle healing after contusion injury in mice reported significantly higher number of regenerating myofibres at the contusion site in groups treated with MSC lines (IM2) with and without scaffold compared with the control group 4 weeks post-injury (2 weeks after treatment), which is consistent with the time point of assessment in the present study [25]. In general, accumulating evidence suggests that application of MSCs facilitate regeneration of myofibres histologically, although the underlying mechanism remains unclear.

There have been several attempts in previous years to determine the mechanisms that underpin the observed superior muscle repair outcomes induced by MSC, but none have been definitive [55,56]. Possible mechanisms by which MSCs contribute to muscle regeneration include direct differentiation into myofibres [57], fusion with existing myofibres with or without differentiation [58,59], and secretion of trophic factors in a paracrine manner that recruit myogenic precursor (satellite) cells and/or attenuate inflammation and fibrosis, thereby creating a conducive regenerative milieu [60,61]. The study by Natsu et al. [48] showed a positive regenerative effect of MSCs on lacerated muscle; however, it failed to demonstrate direct involvement of the injected MSCs in myogenic differentiation, myocyte fusion, or myotube formation. Similarly, studies tracking MSCs after blunt crush injury did not provide evidence of active involvement of the transplanted cells in myogenic differentiation, suggesting that MSCs primarily exert their regenerative action via paracrine effects [21,53]. Indeed, a recent paradigm shift has emerged suggesting that the beneficial effect of MSC may predominantly be attributed to their transient paracrine actions. The role of paracrine action of MSCs in tissue regeneration including skeletal muscle has been previously reviewed [60,62,63]. It has been shown that MSCs release a plethora of trophic, anti-inflammatory, anti-fibrotic and angiogenic factors as well as factors regulating extracellular matrix homeostasis, which are able to modulate the microenvironment, favouring an enhanced tissue regeneration [60,64]. The regenerative potential of MSCs could also be contributed by the immunoregulatory effects of the cells themselves. The physiological response to injury is initiated by the inflammatory phase, which is marked by the infiltration of surrounding as well as circulating macrophages [65]. The surrounding cells may be subjected to a prolonged stress-induced environment that signals for the progenitor satellite cells to produce fibrotic tissues in the subsequent phases of tissue repair [65]. Since MSCs have the ability to reduce the inflammatory response, tissue fibrosis may be reduced sufficiently to prevent excessive scarring thus producing better muscle repair outcomes. We could not ascertain the fate of the injected MSCs, as cell-tracking methods were not employed in this study. Incorporating cell tracking methods in future studies will provide further mechanistic insight into the role of transplanted MSCs in muscle regeneration. Given the relatively short assessment period (7 days post-treatment) and our histological findings of increased population of myoblasts accompanied by reduced fibrotic area in the MSC-treated muscle, it is conceivable that the regenerative effect of MSCs at this stage was primarily mediated through the paracrine mechanism rather than direct differentiation of the cells into myogenic lineage. However, in the absence of in vivo cell tracking and comprehensive profiling of the MSC secretome, this proposition remains speculative at this stage. It has been previously reported that direct injection of MSC-conditioned media into infarcted hearts reduced infarct size as early as 3 days post-treatment, indicating that the beneficial paracrine effect of MSCs can be attained in a relatively short period of time [66]. A previous investigation of MSCs homing after systemic transplantation in a critically ischaemic murine skin flap model demonstrated that MSC aligned along the vascular wall without undergoing endothelial or smooth muscle differentiation, with an accompanying increased in paracrine expression of VEGF and iNOS 4 days after injection [67]. Local injection of MSC extract into a skin defect model increased proliferation of dermal fibroblast, epithelial cells, and vascular endothelial cells, and also enhanced the dermal thickness collagen fibre formation, observed as early as 5 days post-injury [68]. Collectively, these findings further support the view that the paracrine effect of MSCs could manifest within a short time frame. In our study, the histological observation of myoblast aggregation on day 7 post-treatment could potentially be attributed to activation and differentiation of a resident population of muscle progenitors, namely satellite cells, induced by a wide array of soluble bioactive factors released by the injected MSCs either in a direct or indirect manner. The trophic actions of bone marrow-derived MSCs for muscle regeneration have been previously highlighted in a review paper [60]. MSCs are able to modulate the microenvironment surrounding the injured area towards a regenerative state: interleukin (IL)-10, transforming growth factor (TGF)-ß, galectin-1/3, leukaemia inhibitory factor, nitric oxide, and prostaglandin exert immunomodulatory effects; interleukin-6, hepatocyte growth factor, and adrenomedullin promotes favourable extracellular matrix remodelling; and vascular endothelial growth factor, fibroblast growth factor, matrix metalloproteinases, platelet-derived growth factor, and angiopoetin promote angiogenesis, collectively creating a niche conducive for satellite cell survival and activation [60]. MSCs also release stromal derived factor-1, which inhibits apoptosis and directly promotes myogenesis of the tissue-resident progenitor cells. Given the absence of data on the fate of the transplanted MSCs and detailed analyses of the MSC secretome, the present study can only infer an association between MSC treatment and the observed improvements, while the underlying mechanisms remain unclear.

The results of this pilot investigation should be interpreted in light of certain methodological limitations. It is worth noting that conducting a reliable physiological measurement of muscle strength poses technical challenges, which may have contributed to the increased variability of muscle force measurement observed across the groups. Before the actual measurement of tetanic contraction could be obtained in our study, the muscle was subjected to multiple electrical stimulations to optimise the experimental protocol in each animal. This included determination of resting length at which the overlapping of actin and myosin filaments is optimal, incremental stimulation strength until a maximal amplitude of a single muscle twitch was reached, and incremental rise in the frequency of stimulation until a maximal plateau in the amplitude of tetanic contraction was obtained. This procedure was repeated in the contralateral leg. This intricate experimental procedure had the advantage of tailoring the optimisation of tetanic force measurement to the individual animal to ensure recruitment of all motor units in the whole muscle of each animal. Similar approaches have been used in other studies [69,70]. It is notable that most studies used only a single functional parameter to assess muscle function [25,26,29,41,47,49,51]. Although some variation exists in the reporting of muscle contractile function in absolute unit (g [69], N [29,49,51] mN/g [15]); most studies expressed the functional outcome of the regenerated muscle as the ratio of muscle force of the injured to contralateral uninjured leg, which is consistent with our approach. This allows each animal to serve as its own control, which reduces variability caused by inter-animal differences in body size, muscle mass, and baseline muscle strength. In this study, we have attempted to minimise selection bias by randomising the sequence of order of testing between injured and uninjured legs. Nevertheless, it is plausible that even the uninjured muscle could be fatigued when subjected to this lengthy protocol, thereby producing a lesser force. As a precaution, a 15 min interval was introduced between measurements of muscle strength in the injured and uninjured muscles to allow for full recovery and minimise fatigue. Nevertheless, the presence of fatigue could have contributed to a lower tetanic force in the injured compared with the contralateral muscle, although this effect was consistent across groups. Another limitation that is worth acknowledging is the lack of assessment of additional functional parameters of muscle function such as endurance or recovery rate after fatigue. A more comprehensive evaluation of muscle morphometrics and composition (lean muscle mass, myofibre cross-sectional area) as well as contractile performance (endurance testing ex vivo, contraction dynamics, and force-velocity analysis) [71,72] would be valuable in future studies to better characterise the impact of MSC treatment on muscle regeneration.

While closed muscle injuries such as bruising or stretching are more commonly seen clinically, there remains a significant unmet need to improve outcomes in open laceration injuries, where current treatments often yield inconsistent results. Clinically, surgical intervention can improve functional recovery to some extent but often fails to fully restore normal muscle function, with recovery periods frequently prolonged. This is supported by a previous pre-clinical study, which reported that surgical repair of gastrocnemius muscle laceration achieved only 81% recovery of muscle strength at 1-month post-injury, compared with 35% following conservative treatment; outcomes were even poorer (18%) when muscles were immobilised during healing [73]. Prolonged recovery times are clinically undesirable as this increases the risk of disuse atrophy and delays return to normal activity or work. The likelihood of regaining pre-injury functional status is closely related to recovery duration, and immobilised patients may lose up to 40% of their muscle strength within just one week [74], which likely explains the poor functional outcomes reported in the aforementioned preclinical study [73]. The ability of MSC therapy to restore contractile performance to near-intact levels within two weeks as shown in our study suggests its potential to accelerate functional recovery after traumatic muscle injuries. Clinically, this could shorten rehabilitation time, improve mobility, and reduce long-term deficits such as chronic weakness. Our findings, although preliminary, suggest that MSCs could support muscle healing following surgery and may serve as at least an adjuvant to surgical intervention. However, validating this potential requires larger, more robust studies, including mechanistic investigations into their mode of action.

## 5. Conclusions

This pilot study provides preliminary evidence that local treatment with allogeneic MSC is associated with functional recovery of lacerated skeletal muscle within a relatively short time frame, although the underlying mechanisms remain undetermined. These findings serve as an important basis for future research exploring the therapeutic potential of MSCs in muscle regeneration. With further research confirming the positive outcomes, MSCs may potentially facilitate earlier recovery of functional deficit, enabling a quicker return to normal activity after severe muscle injury. However, larger and mechanistically focused investigations with more comprehensive multimodal assessment of muscle function supported by an extensive exploration of its structural integrity are warranted to validate these findings before the potential clinical utility of MSCs can be realised.

## Figures and Tables

**Table 1:** Comparison of tetanic contraction force, expressed as weight displacement in grams (g), between the right and left leg intact gastrocnemius muscles and relative muscle force of the right versus left intact legs.

Specimen No.	Muscle Force (g) in the Right Leg	Muscle Force (g) in the Left Leg	Ratio of Muscle Force in the Right Relative to the Left Leg (%)
1	200.3	182.1	110.0
2	118.2	122	96.9
3	194.5	193.1	100.7
4	127.6	120.8	105.6
5	151.9	176	86.3
6	186.9	161.4	115.8
Mean	163.2 ± 35.6	159.2 ± 31.0	102.6 ± 10.4

**Table 2:** Comparison of mean tetanic contraction force, measured as weight displacement (g), and the mean ratio of the injured (right) normalised to the contralateral uninjured (left) gastrocnemius muscle (internal control) among the study groups on day 7 post-treatment (3 weeks post-injury).

Study Group (6 Subjects per Group)	Force of Tetanic Muscle Contraction ± SD (g)		Ratio of Muscle Force of Injured Relative to Contralateral Uninjured Muscle (R/L leg) (%)
	Injured Muscle (Right Leg)	Uninjured Muscle (Left Leg)	
No treatment	162.7 ± 53.9	195.1 ± 33.1	83.1 ± 14.7
Saline-treated	121.4 ± 44.5	158.7 ± 45.5	76.7 ± 4.6
MSCs-treated	180.5 ± 27.2	187.2 ± 19.0	96.8 ± 15.0
	**Uninjured Muscle**		
	**Right Leg (R)**	**Left Leg (L)**	**Ratio R/L (%)**
Intact muscle	163.2 ± 35.6	159.2 ± 31.0	102.6 ± 10.4

**Table 3:** Comparison of mean force of tetanic contraction measured as weight displacement (g) and mean ratio of the injured (right) normalised to the contralateral uninjured (left) gastrocnemius muscle (internal control) among the study groups on day 14 post-treatment (1-month post-injury).

Study Group (6 Subjects per Group)	Force of Tetanic Muscle Contraction ± SD (g)		Ratio of Muscle Force of Injured Relative to Contralateral Uninjured Muscle (R/L leg) (%)
	Injured Muscle (Right Leg)	Uninjured Muscle (Left Leg)	
No treatment	180.2 ± 24.2	204.7 ± 6.8	88.1 ± 3.41
Saline-treated	114.4 ± 46.1	147.8 ± 56.7	78.4 ± 6.47
MSCs-treated	192.8 ± 39.6	178.6 ± 36.0	110.8 ± 6.46
	**Uninjured Muscle**		
	**Right Leg (R)**	**Left Leg (L)**	**Ratio R/L (%)**
Intact muscle	163.2 ± 35.6	159.2 ± 31.0	102.6 ± 10.4

**Table 4:** Quantitative analysis on Masson’s Trichrome-stained histological sections to determine fibrosis index in injured muscles treated with saline and MSCs for six biological samples.

Treatment Group	Mean Blue/Red Intensity Ratio	SD	Fibrosis Index (%)	SD	*p* Value
Saline	0.865	0.032	31.77	0.43	<0.0001
MSCs	0.779	0.014	29.30	0.29	<0.0001

## Data Availability

The original contributions presented in this study are included in the article. Further inquiries can be directed to the corresponding authors.

## Abbreviations

BM-MSCs	Bone marrow-derived mesenchymal stem cells
H	Hour
H&E	Hematoxylin and Eosin
MSCs	Mesenchymal stem cells
Hz	Hertz
PBS	Phosphate-buffered solution
SD	Standard deviation
